# Successful treatment of pan-drug resistant *Acinetobacter baumannii* meningitis/ventriculitis following craniotomy and external ventricular drainage: a case report

**DOI:** 10.1093/jscr/rjae603

**Published:** 2024-09-23

**Authors:** Aleksandra Dimovska Gavrilovska, Hristijan Veljanovski, Radomir Jovchevski

**Affiliations:** University Clinic of Neurosurgery, Clinical Center “Mother Theresa”, 1000 Skopje, Republic of North Macedonia; University Clinic of Neurosurgery, Clinical Center “Mother Theresa”, 1000 Skopje, Republic of North Macedonia; Institute of Microbiology and Parasitology, Faculty of Medicine, Ss. Cyril and Methodius University in Skopje, 50 Divizija 6, 1000 Skopje, Republic of North Macedonia

**Keywords:** central nervous system infection, colistin, intraventricular

## Abstract

Healthcare-associated central nervous system infections are a significant complication for patients undergoing neurosurgical interventions. We present a case of a 6-year-old patient with an embryonal tumor of the central nervous system. Following a craniotomy for the resection of the tumor, an external ventricular drainage was placed. Several weeks after surgery, she developed signs of meningism. Cerebrospinal fluid cultures were positive for pan-drug resistant *Acinetobacter baumannii*. Several revisions with the insertion of new external valves were done. She was treated with intravenously meropenem and vancomycin combined with colistin administrated intraventricularly. Significant improvement was seen clinically with negative cultures after 2 weeks. The synergistic action of colistin administrated locally combined with systemic antibiotics may be a promising option for critically ill patients with pan-drug resistant *A. baumannii* central nervous system infection.

## Introduction

Hospital-acquired infections of the central nervous system (CNS) (meningitis and ventriculitis) are significant complications of neurosurgical patients undergoing craniotomy, mainly when associated with resistant microorganisms [1]. These infections are up to 8% in patients with external ventricular drainage systems. Out of gram-negative rods, *Acinetobacter baumannii* has an incidence of 3.6%–11.2% of cases of healthcare-associated meningitis [2]. Treatment is complicated by the rise of antimicrobial resistance, especially for meropenem and colistin [3]. This case highlights the successful eradication of *A. baumannii* infection following the addition of intraventricular colistin.

## Case report

**Figure 1:**
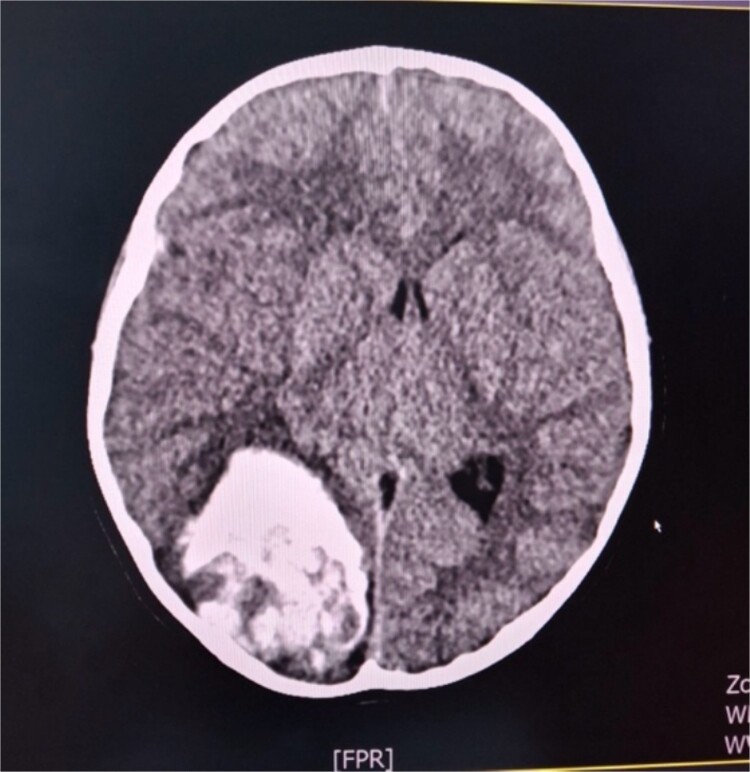
Axial cerebral CT scan shows massive hemorrhaging tumor in the right occipital region with perifocal oedema.

A 6-year-old female child in a comatose state with two dilated pupils as an emergency case was brought to the University Clinic of Neurosurgery. A cerebral CT scan showed a hemоrrhagic voluminous tumor in the right occipital region (Fig. 1). Immediately, she underwent a craniotomy for maximum tumor reduction. During postoperative days, she was stable, a control cerebral MRI scan with contrast was done, and a second surgery for tumor reduction was decided to be done. The findings of the histopathologic examination of the tumor were consistent with an embryonal tumor of CNS NEC/NOS (not elsewhere classified/non-otherwise specified) (CNS WHO grade 4). A spinal tap was performed, and the microbiological findings of the cerebrospinal fluid cultures were negative. Cerebrospinal fluid cytology was within normal limits. She was referred to a pediatric oncologist. After the first cycle of chemotherapy, she developed signs of hydrocephalus and meningism. The patient underwent external ventricular drainage with the insertion of a middle-pressure valve.

**Figure 2:**
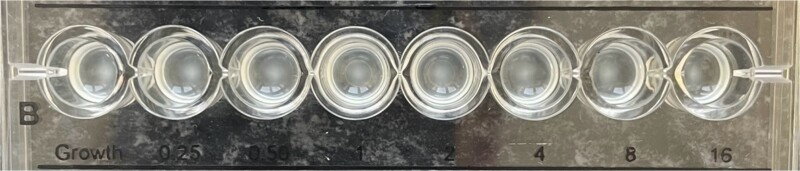
Colistin susceptibility by broth microdilution. Resistant isolate (MIC = >16 mg/L).

**Figure 3:**
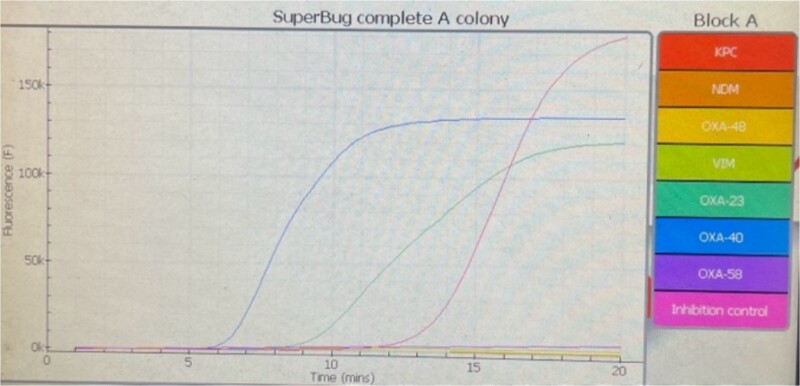
Amplification and anneal curves of amplified products of OXA-23 and OXA-40 genes of *Acinetobacter baumannii*.

Several days postoperative, cytologically, cerebrospinal fluid was cloudy with elevated values of lactates and albumin. Cytopathological examination of cerebrospinal fluid smears detected numerous polymorphonuclear leucocytes in a fibrin network consistent with acute purulent meningitis, single lymphocytes, and scarce epithelial cells with mild atypia. On three occasions, cerebrospinal fluid cultures were positive for *A. baumannii.* Isolates were non-susceptible to all agents in all antimicrobial categories. Standard disk diffusion testing method guidelines by EUCAST (The European Committee on Antimicrobial Susceptibility Testing) were used to test susceptibility to frequently prescribed antibiotics. Commercial antibiotic disks (Oxoid, UK) that were used were: imipenem (10 μg), meropenem (10 μg), gentamicin (10 μg), amikacin (30 μg), ciprofloxacin (5 μg), levofloxacin (5 μg), and trimethoprim-sulfamethoxazole (25 μg). Susceptibility for colistin was done with a broth microdilution test (Liofilchem, Italy) according to EUCAST guidelines. Minimal inhibitory concentration (MIC) was >16 mg/L (Fig. 2). Simultaneously, antimicrobial susceptibility was done automatically by using the VITEK-2 Compact system (Biomerieux, France). MICs for meropenem and colistin were >8 mg/L. *A. baumannii* isolates underwent PCR analysis to determine the presence of specific carbapenemase genes (bla_oxa_ genes). We detected bla_oxa40_ and bla_oxa23_ genes (Fig. 3). Meropenem (500 mg every 8 h) and vancomycin (600 mg every 8 h) were administrated intravenously as systemic therapy.

**Figure 4:**
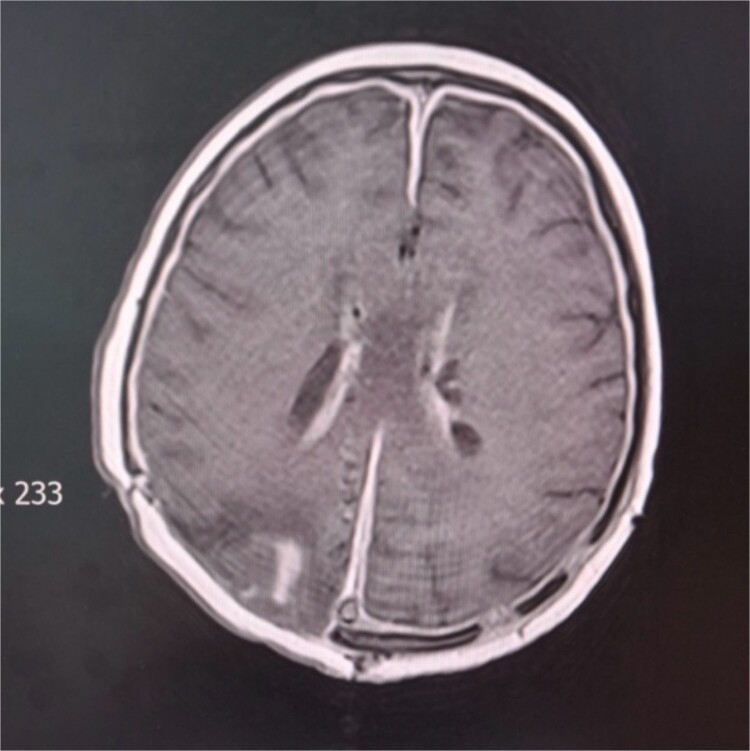
Cerebral MRI. Axial T1 weighted MRI showing ventriculitis with two external ventricular drainages (EVD) in the frontal horns of lateral ventricles.

During the hospital stay, the valve became blocked, so another revision was done by inserting a new valve. The next day, re-occlusion of the valve occurred. Consequently, the whole external drainage system was removed. An external drain without a pressure valve was inserted in the right ventricle’s ventral horn. The patient started to develop symptoms of increased intracranial pressure, nausea, and vomiting. A control cerebral CT scan showed unilateral hydrocephalus; therefore, in the ventral horn of the left lateral ventricle, a second external drain without a valve was inserted. The patient started to become febrile with peaks of temperature to 38.5°C. T1-weighted gadolinium-enhanced MRI studies revealed ventriculitis (Fig. 4). Intraventricular colistin sulfate was added to the antibiotic treatment at 200.000 IU daily in each ventricle for 2 weeks. The ventricular drainage device was closed for 30 min after the injection of the antibiotic. In several instances, cerebrospinal fluid was collected from external drains for cytological and microbiological evaluation during this period. Cytological parameters tended to decrease until the normalization of the elements. On five different occasions, cerebrospinal fluid cultures were sterile. She was discharged from our clinic in stable general and neurological condition, afebrile and conscious. The patient is currently undergoing proton radiation therapy in a hospital abroad.

## Discussion

We detailed the clinical presentation and treatment outcome of a single neurosurgical patient with *A. baumannii* infection. This case highlighted an unusual characteristic: a pan-drug-resistant isolate does not necessarily mean an unfortunate result.

Postoperative CNS infections are severe complications following cranial surgery. The cerebrospinal external fluid drainage system is a health risk for infections, especially those caused by *A. baumannii*. The drainage tube is a potential source of bacteria getting into the brain [4].

The choice and kinetics of antimicrobial agents are crucial in CNS infections because the blood–brain barrier (BBB) has unique characteristics that limit the passage of molecules [2]. Penetration of colistin and, consequently, concentration in cerebrospinal fluid is low. Therefore, intraventricular administration can result in a distribution in the hole cerebrospinal fluid space with concentrations above the isolate’s MIC [5].

In a study by Liang *et al*. [4], seven patients received intraventricular antibiotic agents, and the CSF sterilization rate was 71.4%.

Abdallah *et al.* [3] demonstrated in their cases that a combination of intravenous and intraventricular administration of antibiotics achieves good therapeutic outcomes. One of the most common resistance mechanisms to carbapenems is their enzymatic hydrolysis mediated by β lactamases (carbapenemases). OXA β lactamases are class D enzymes. The first OXA enzyme identified in *A. baumannii* was OXA-23, and the first representative from the OXA-24/40-like group was OXA-40 [6]. In our case, we identified bla_oxa40_ and bla_oxa23_ genes in the isolates.

Although *A. baumannii* is resistant to meropenem and colistin, the combination of meropenem and colistin sulfate has a potential synergistic effect on this bacterium [7]. Meropenem with vancomycin should also be chosen as a systemic antibiotic treatment [8]. In a study by Yang *et al.* [9], vancomycin and meropenem were selected as the initial empirical treatment. In this case, our patient responded to a combination therapy that included intravenously administered meropenem, vancomycin, and intraventricularly colistin.

## Conclusion

We demonstrated our case of pan-drug resistant *A. baumannii* infection successfully treated with locally administered colistin in combination with systemic therapy. Therefore, this case suggests that intraventricularly applied colistin can be an excellent therapeutic option against meningitis/ventriculitis caused by *A. baumannii.*
